# “Actor-critic” dichotomous hyperactivation and hypoconnectivity in obsessive–compulsive disorder

**DOI:** 10.1016/j.nicl.2024.103729

**Published:** 2024-12-31

**Authors:** Ana Araújo, Isabel C. Duarte, Teresa Sousa, Sofia Meneses, Ana T. Pereira, Trevor Robbins, António Macedo, Miguel Castelo-Branco

**Affiliations:** a Coimbra Institute for Biomedical Imaging and Translational Research (CIBIT), University of Coimbra, 3000-548 Coimbra, Portugal; b Institute for Nuclear Sciences Applied to Health (ICNAS), University of Coimbra, 3000-548 Coimbra, Portugal; c Institute of Psychological Medicine, Faculty of Medicine, University of Coimbra, 3004-504 Coimbra, Portugal; d Faculty of Medicine, Institute of Physiology, University of Coimbra, 3004-531 Coimbra, Portugal; e Department of Psychiatry, Local Health Unit of Coimbra, 3004-561 Coimbra, Portugal; f Department of Psychology, Local Health Unit of Coimbra, 3004-561 Coimbra, Portugal; g Department of Psychology, Behavioural and Clinical Neuroscience Institute, University of Cambridge, Cambridge CB2 3EB, UK

**Keywords:** Obsessive–compulsive disorder, Actor-critic, Caudate, Putamen, Ventral tegmental area, Inhibition, Error monitoring

## Abstract

Dysfunctional response inhibition, mediated by the striatum and its connections, is thought to underly the clinical manifestations of obsessive–compulsive disorder (OCD). However, the exact neural mechanisms remain controversial. In this study, we undertook a novel approach by positing that a) inhibition is a dynamic construct inherently susceptible to numerous failures, which require error-processing, and b) the actor-critic framework of reinforcement learning can integrate neural patterns of inhibition and error-processing in OCD with their behavioural correlates. We invited nineteen adults with OCD and 21 age-matched healthy controls to perform an fMRI-adjusted stop-signal task. Then, we extracted brain activation and connectivity values regarding distinct task phases in the “actor” and “critic” regions, here corresponding to the caudate’s head and dorsal putamen, and midbrain’s nuclei (ventral tegmental area and substantia nigra). During response preparation phases of the inhibitory process, individuals with OCD exhibited decreased functional connectivity between the “critic” structures and frontal regions involved in cognitive and executive control. Activity analysis revealed task-related hyperactivation in the midbrain alongside error-processing-specific hyperactivation in the striatum, which was correlated with excessive behavioural slowness, also found in the clinical group. Finally, we identified a remarkable opponency between activity in the ventral tegmental area and caudate leading to direct increases and indirect decreases in symptom severity. We propose a unique “actor-critic”-based domain- and timing-dependent neural profile in OCD, reflecting “harm-avoidant” styles for response suppression, and influencing symptom severity. The dichotomy of hypoconnectivity and hyperactivation in the “critic” along with the opponent relationship between the “actor” and the “critic” in determining symptom severity suggests the implication of neural adaptation mechanisms in OCD with potential relevance for neurobiologically-driven therapies.

## Highlights

• Adults with OCD used an inhibition strategy marked by overcautious tendencies.
• The OCD group exhibited hyperactivation and hypoconnectivity in actor-critic regions.
• Caudate hyperactivation during error processing correlated with excessive slowness.
• Midbrai overeractivation led to direct increases and caudate-mediated decreases in OCD symptoms.

## Graphical abstract

**Figure:**
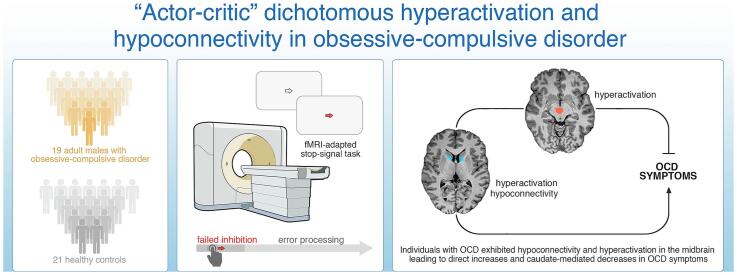

## 1 Introduction

Obsessive-compulsive disorder (OCD) has been conceptualized as a dysfunction in behavioural inhibition (Robbins et al., 2012, de Wit et al., 2012), underpinned by imbalanced cortical-striatal activities (Banca et al., 2015, Milad and Rauch, 2012, Robbins et al., 2019), with the striatum serving as a central node (Baxter et al., 1992, Menzies et al., 2008, Pauls et al., 2014, Whiteside et al., 2004). However, neuroimaging studies have reported inconsistent activation patterns in the caudate and putamen of individuals with OCD during inhibition tasks, including increased (Morein-Zamir et al., 2016, Yücel et al., 2007), decreased (Kang et al., 2013, Nakao et al., 2005, Page et al., 2009), and unchanged activation (Roth et al., 2007, Schlösser et al., 2010). Connectivity between regions subserving inhibition appears to be reduced (Banca et al., 2015, Hampshire et al., 2020, Kim et al., 2024, Vaghi et al., 2017a, Vaghi et al., 2017b), interfering with the behavioural strategies employed by affected individuals (Van Velzen et al., 2015).

Phenotypically, OCD manifests through excessive self-monitoring, “harm-avoidant” tendencies, and repetitive behaviours (Abramovitch et al., 2012, Salkovskis, 1999). The prominent maladaptive habit hypothesis (Voon et al., 2015) posits that an imbalance between goal-directed (medial prefrontal cortex and caudate nucleus) and habitual systems (putamen and motor cortical regions) is the basis for the repetition of dysfunctional behaviours in this disorder (Banca et al., 2015). Nonetheless, while habitual learning can explain the reinforcement of compulsions (Sakai et al., 2022), it does not adequately address other OCD core features, such as obsessions (Abramovitch et al., 2012, Salkovskis, 1999). Moreover, it remains unclear whether excessive habit formation results from a difficulty in inhibiting habits, stronger habit learning, or impairment in goal-directed control (Gillan et al., 2016, Gillan and Robbins, 2014, Robbins et al., 2019). In the same vein, the co-occurrence of seemingly opposing characteristic – being “harm avoidant” while failing to accurately monitor their own behaviour – in individuals with OCD poses significant challenges to the comprehensive integration of neurobiological evidence with their behavioural counterparts. Are response inhibition processes and associated psychobiological phenomena hypo- or hyperfunctional in OCD?

Answering this question may require new ecological approaches to inhibition. In real-life situations, inhibition, as a trade-off of being highly efficient, is frequently prone to numerous failures that necessitate error monitoring, learning, and behavioural adjustment (Balleine and O’Doherty, 2010, Bari and Robbins, 2013). In contrast, most neuroimaging techniques tend to conflate distinct components into a general process (Chevrier & Schachar, 2010), leading to interpretations that reflect the sum or result of related but potentially distinct temporal subprocesses (Ljungberg et al., 1991). This artificial aggregation undermines the precise identification of the impairments that are uniquely involved in particular neuropsychiatric disorders (Chevrier and Schachar, 2010, Morein-Zamir et al., 2016). Conversely, OCD studies that dissect inhibitory task requirements tend to identify specific changes in error-related processes (Berlin and Lee, 2019, Endrass et al., 2010, Fitzgerald et al., 2005, Gehring et al., 2000, Morein-Zamir et al., 2016, Norman et al., 2019). Similarly, when exposed to symptom-provoking conditions, individuals with OCD exhibit a combination of hyperactivation succeeded by deactivation in the putamen during “exposure” and “relief” events, respectively (Banca et al., 2015). All these findings suggest that the ever-changing environmental context modulates neural response and behavioural patterns, and therefore, should be more explicitly considered in research designs.

Accordingly, we postulated that a) inhibition can be envisaged as an ecological construct encompassing error monitoring and learning (Bari & Robbins, 2013), b) neural and behavioural inhibition unfold across sequential phases (Chevrier & Schachar, 2010), c) OCD may affect specific inhibition-related subprocesses and/or phases, particularly those associated with errors (Morein-Zamir et al., 2016, Norman et al., 2019), and d) the actor-critic framework of reinforcement learning effectively incorporates the main dynamic and multilevel features underlying inhibitory processes (Araújo et al., 2024, Barto, 1995, Sutton and Barto, 1999).

The actor-critic models (Barto, 1995, Sutton and Barto, 1999) posit that learning relies on the inter-relationships between a policy-based (the “actor”) and a value-based (the “critic”) module. The “actor” explores the action space, developing and implementing a policy to select actions that maximize the weighted sum of future rewards. Concurrently, the “critic” estimates this sum by comparing its reward prediction with the actual outcome, calculating a prediction error. The prediction error guides the “actor” towards actions that yield higher expected returns by updating its policy based on the most recent action. Given the similarities between the actor-critic interplay and both human (e.g., Ballard et al., 2011, D’Ardenne et al., 2008, O’Doherty et al., 2004, Schönberg et al., 2007) and animal (e.g., Balleine and Dickinson, 1998, Fiorillo et al., 2008, Keiflin et al., 2019, Matsumoto and Hikosaka, 2009, Mohebi et al., 2019, Schultz et al., 1997, Solié et al., 2022, Suri and Schultz, 1999) behaviour, the actor-critic model has been extensively employed to investigate the structure–function relationships in specific brain areas, particularly the basal ganglia and dopaminergic midbrain nuclei. Our previous study in healthy humans (Araújo et al., 2024) confirmed the involvement of neural circuits linking the dorsal parts of the caudate and putamen with the lateral ventral tegmental area (VTA) and substantia nigra (SN) during inhibition errors, where these regions exhibited a neurobehavioural profile consistent with the “actor” and “critic” roles.

Building upon these findings, the present study aims to investigate the role of the “actor-critic” brain regions during response inhibition in individuals with OCD. We employed a functional magnetic resonance imaging (fMRI) approach to isolate the neural correlates of successful inhibition, failed inhibition, and post-failed inhibition processing (henceforth termed error-processing) during the stop-signal task (STT) (Logan and Cowan, 1984, Verbruggen et al., 2019, Verbruggen and Logan, 2008). The SST is a classical paradigm for assessing response inhibition, specifically the termination of an already initiated action (action cancellation). As the task inevitably generates numerous errors, it also involves error monitoring (Aron et al., 2014) within a reinforcement learning pathway, as previously demonstrated (Araújo et al., 2024, Chevrier and Schachar, 2010). Participants are instructed to respond to go-signals but to refrain from responding on a minority of trials if a stop-signal follows a go-signal. We defined inhibition errors as those occurring during failed stop-trials, where the subject should have withheld the response but inadvertently pressed the button. The task algorithm is adaptive to keep an approximate 50 % error level (Verbruggen et al., 2019), and participants were informed of this. They were also apprised that both responding fast on go-trials and inhibiting the button press on stop-trials were equally important, being encouraged to discover the optimal balance between rapid response execution and successful inhibition (“speed-accuracy trade-off”) (Verbruggen et al., 2019). While fast suppression of all ongoing actions on stop-trials is thought to recruit the subthalamic nucleus via the hyperdirect pathway of the basal ganglia, strategic response slowing may involve proactive competition between direct and indirect striatal pathways (Jahanshahi et al., 2015).

Our primary hypothesis focused on the utility of the actor-critic configuration as a comprehensive rationale for elucidating OCD-specific behavioural strategies and dynamic neural responses during inhibition. We hypothesized that individuals with OCD would prioritize error avoidance (successful inhibition) at the expense of excessive response slowing, thereby disrupting the “speed-accuracy trade-off” during the SST. In line with this, we anticipated observing hyperactivation in the striatum and midbrain during error-processing, corresponding to the period when subjects recognize that an error has occurred. Regarding task-related functional connectivity, we predicted reduced connectivity between the “actor-critic” network and other brain regions, particularly in circuits involving mesocorticolimbic and nigrostriatal structures. Ultimately, we aimed to test a hypothetical framework of the interplay between neural pathways representing the “actor” and the “critic”, leading to OCD symptom manifestations.

## 2 Materials and methods

### 2.1 Participants

Forty right-handed adult males divided into two age-matched groups – HC subjects (*n* = 21) and OCD patients (*n* = 19) (28.67 ± 9.03 *versus* 31.68 ± 9.35 years; *t*(38) = −1.038, *P* = 0.306) – were included in the present analyses. To maximize the homogeneity of our groups in the network activation underlying the SST (Rubia et al., 2013) and due to recruitment constraints, only male participants were recruited. The age of the participants was 30.10 ± 9.190 years (*mean* ± *standard deviation*), allowing us to evaluate a neurodevelopmental period in which inhibitory abilities are already well-developed (Rubia et al., 2013). For detailed information on the participants’ characteristics, please see Supplementary Table 1.

The participants from the OCD group were recruited from the OCD Treatment Unit of the Coimbra Hospital and University Centre. Clinical diagnosis of OCD followed the criteria of the *Diagnostic and Statistical Manual of Mental Disorders, 5th edition*, applied by an experienced psychiatrist and psychologist, as part of the Unit’s usual clinical practice. To evaluate symptom severity, the first author (AA) applied the Portuguese-validated translation of the *Yale-Brown Obsessive Compulsive Scale – Second Edition (Y-BOCS-2)* (Castro-Rodrigues et al., 2018, Storch et al., 2010). Mean *Y-BOCS-2* scores were 25.26 ± 5.84, which corresponds to moderate to severe symptom grades. Subjects from the HC group were recruited locally using social media advertising.

Exclusion criteria for both groups included medical or psychiatric disorders (except OCD for the OCD group) affecting brain development (e.g., epilepsy, attention-deficit/hyperactivity disorder, neurological or genetic conditions), intellectual disability, drug/alcohol or behavioural dependencies, history of severe head injury, abnormal structural MRI scans, and MRI contraindications. For inclusion in the OCD group, past diagnoses or subclinical symptoms of disorders related or secondary to OCD were allowed. All participants in the OCD group, except one, were medicated with antidepressants, and a minority were also prescribed antipsychotics, lamotrigine, and/or memantine. Although initially enrolled in the study, one participant with OCD was excluded because he showed signs of severe depression that, at the time of the evaluation, was autonomous from OCD symptoms. We also excluded two participants from the HC group, one because he showed signs of attention-deficit/hyperactivity disorder and the other because of a head injury not reported in the initial contact for the recruitment. The exclusion of those individuals resulted in a total of 40 participants.

All participants underwent a comprehensive clinical assessment including an interview with the *Mini-International Neuropsychiatric Interview (MINI) – Portuguese version 5.0.0 for DSM-IV* (Lecrubier et al., 1992). Evaluation with the *Wechsler Adult Intelligence Scale – third edition (WAIS-III)* (Wechsler, 1997, Wechsler, 2008) was applied to all except one participant of the HC group and three participants of the OCD group who were unable to return to the research center until the end of the recruitment. Those participants were, however, included in the analysis because they showed no signs of altered intelligence quotient (IQ) and were able to comprehend the task. Minimum full-scale IQ scores were 106, in the HC group, and 80, in the OCD group. Although the relationship between inhibitory control and intelligence remains unclear (Michel and Anderson, 2009), we assumed that IQ would not affect task performance and brain activation, as indicated by our previous work using the SST (Araújo et al., 2024).

This study was conducted under the Declaration of Helsinki. We obtained ethical approval from the local Research Ethics Committee (CHUC-089-20), and all participants signed written informed consent after a detailed explanation of the study procedure. Evaluation sessions were scheduled according to the clinical appointments at the hospital.

### 2.2 MRI procedure

The acquisition session comprised one structural and three functional sequences. The task was performed during the last half-hour of an MRI session with a total duration of 1 h. The SST was presented on an LCD monitor (48.5 × 87.8 cm, 1920 × 1080-pixel resolution, 60 Hz refresh rate) which the participants viewed through a mirror mounted above their eyes. The distances from the eye to the top and to the bottom of the screen were around 1750 mm and 1825 mm, respectively. Participants' responses were collected via an MRI-compatible response box. Participants used the dominant hand, which was the right for the entire sample. When necessary, we ensured correction to normal vision using specific magnetic field-compatible eyeglasses.

### 2.3 Stop-signal task

**Fig. 1:**
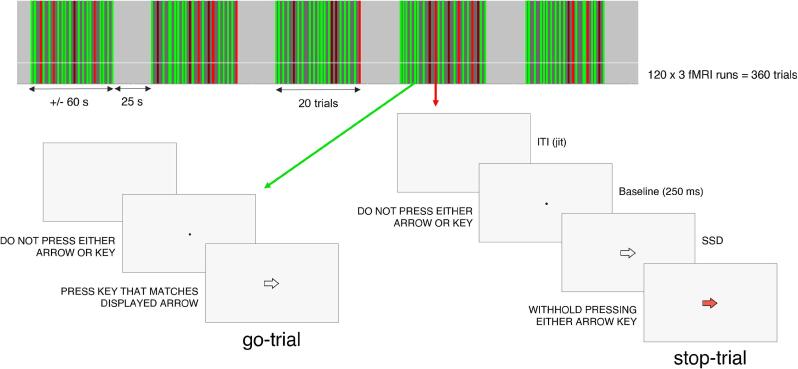
Schematic of the stop-signal task. The task included 3 runs of 120 trials (a total of 360 trials). Within each run, the trials were presented in blocks of 20 repetitions (60 s) interleaved with a baseline/response preparation period of 25 s. Go-trials (75 %) began with a fixation dot (250 ms) followed by a go-signal, which was a white arrow either pointing to the right or left side on the screen, instructing participants to press the left or right button of the response box. Stop-trials (25 %) began with a fixation dot (250 ms) followed by the white arrow, which turned red (stop-signal) after a variable period of time (stop-signal delay), instructing the subject to withhold the response. Approximately half of the stop-trials could not be stopped, as a result of the staircase implementation. The inter-trial interval (time between the end of the previous trial and the start of the current one) was jittered between 750 and 2750 ms. ITI: inter-trial interval; jit: jittered; SSD: stop-signal delay. (For interpretation of the references to colour in this figure legend, the reader is referred to the web version of this article.)

We developed and presented the SST using Psychophysics Toolbox 3 on MatLab R2019b (The MathWorks, Inc., USA) according to previous guidelines (Li et al., 2006, Logan and Cowan, 1984, Verbruggen et al., 2019), as illustrated in Fig. 1. We asked participants to respond by pressing one of two buttons (left or right) of the response box. They were instructed that a) they should quickly press the button on go-trials (75 % of the total number of trials) and withhold the response on stop-trials (25 % of the total number of trials, randomly presented); b) both stopping on stop-trials and responding fast on go-trials were equally important; c) go-trials would be aborted after a fixed period of time; d) the task was adaptive to their performance so that at the end of each run the number of *Successful* and *Failed Stop* was approximately equal, and e) stopping would not be possible in some of the stop-trials. Participants completed 3 runs of 120 trials (a total of 360 trials: 270 go + 90 stop trials). Within each run, blocks of 20 trials were interleaved with baseline periods of 25 s, corresponding to response preparation phases. We randomly jittered the mean *Inter-trial Interval* (ITI; the time between the end of the previous trial and the start of the current trial) between 750 and 2750 ms to optimize statistical efficiency. The trial began with a black dot (250 ms) followed by an arrow pointing to the right or left side on the screen (go-signal). The arrow was displayed within the central 2.25 degrees of the visual field. In 25 % of trials (the stop trials), the arrow in the go-signal turned red (stop-signal). The trials ended at the button press or after 1250 ms if the participant did not respond (*Successful Stop* or *Omission Go*). The stop-signal delay (the delay between a go-signal and a stop-signal) started at 200 ms and was dynamically changed according to an adaptive staircase procedure, with a 50 % performance criterion. If the participant stopped successfully on a stop trial, the stop-signal delay latency of the following stop trial increased by 50 ms (up to a maximum of 950 ms), making the task more difficult for the next trial; if the participant failed, the stop signal delay latency decreased by 50 ms (up to a minimum of 100 ms). In this way, at the end of the task, successful stopping was always approximately 50 %. Feedback about task performance appeared on the screen at the end of each run. Before entering the MRI scanner, participants performed a training session (240 trials) of the task.

### 2.4 fMRI acquisition and preprocessing

We acquired functional MRI images using a 3 Tesla Magnetom Prisma Fit scanner (Siemens, Erlangen, Germany), with a 64-channel head coil. The scanning session included one T1-weighted 3D anatomical magnetization prepared rapid acquisition gradient echo pulse sequence, with a repetition time (TR) = 1000 ms, echo time (TE) = 3.5 ms, resolution 2 mm3, flip angle = 7°, 192 slices and field of view (FOV) = 256 × 256 mm. Afterward, we acquired three functional runs using a T2*-weighted gradient echo-planar imaging sequence, with a multi-band acceleration factor of 6, a slice thickness of 2 mm and voxel size 2 mm^2^, 72 interleaved slices without gap, phase encoding direction from anterior to posterior, parallel to the AC-PC line, TR = 1000 ms, TE = 37 ms, flip angle of 68° and FOV of 200 × 200.

Data pre-processing was performed on BrainVoyager 22.0 and 22.4 software (Brain Innovation, Maastricht, The Netherlands). Pre-processing and data analysis were very similar across the two versions so the procedure reliability was not affected. Pre-processing included slice-scan time correction, 3D head-motion correction, and temporal high-pass filtering (2 cycles per run). To correct for geometrical distortion, we used the COPE plugin (Breman et al., 2020). Then, we co-registered the resulting fMRI data and anatomical T1-image, applied a normalization to Montreal Neurological Institute (MNI) standard atlas, and performed spatial smooth using a Gaussian kernel with FWHM of 4 mm. Runs exceeding 6 mm of movement in any axis were excluded from further analysis. Accordingly, we excluded a total of 12 runs (9 in the HC subgroup, and 3 in the OCD group). We added motion and physiological signals (respiration and cardiac signals) as confound predictors into the GLM model. PhysIO toolbox for SPM in Matlab (Frässle et al., 2021, Kasper et al., 2017) was used to create the physiological confound predictors.

### 2.5 Stop-signal task performance variables

To characterize group performance (Logan and Cowan, 1984, Verbruggen and Logan, 2008, Verbruggen et al., n.d., Verbruggen and Logan, 2008), we calculated the mean reaction time on go trials (*Go RT*), mean reaction time on trials after failed stop (*Post-error RT*), number of non-responses on go trials in relation to the number of total trials (*Omission Go*), probability of stopping on stop-trials (Successful stop), and the stop-signal reaction time (*SSRT*). To estimate the *SSRT*, we used the integration method from Verbruggen et al. (2019), taking into consideration the independent race model (Logan and Cowan, 1984). The *SSRT* is a quantitative estimate of the time needed to abort a prepotent (already initiated) response, which reflects the overall efficiency of the stopping process (Verbruggen and Logan, 2008). For the present analysis, we were interested in capturing the individuals’ behavioural strategies in response to errors, so we used the *Go RT* and *Post-error RT* (Berlin and Lee, 2019). Higher reaction times are associated with slower responses, usually applied to increase the likelihood of successful stopping (“speed-accuracy trade-off”) (Verbruggen and Logan, 2008). Considering our hypothesis that OCD patients would apply a distinct inhibitory strategy, for inclusion in the present analysis, we used a threshold of *Omission Go* trials <15 %, which is more liberal than the proposed 5 % for healthy participants (Verbruggen et al, 2019).

### 2.6 Data analysis

#### 2.6.1 Brain activity

To analyse functional neuroimaging data, we used the Brain Voyager software, versions 22.0 and 22.4 (Brain Innovation, Maastricht, the Netherlands). In the first-level analysis for each subject, we performed a GLM approach. We obtained the predictor’s model by convolution of the time course of each condition with a two-gamma hemodynamic response function and modeled all regressors with a duration of 1 TR (1 s) from the onset of the stimulus presentation (left and right-pointing arrow). According to recent consensus work (Verbruggen et al., 2019) and our hypotheses, five types of trial outcomes were included as predictors in our model: *Correct Go* (go trials in which participants pressed the correct right or left button), *Successful Stop* (stop trials in which participants inhibited the response of pressing the button), *Failed Stop* (stop trials in which participants incorrectly pressed the button), *Post-error-processing* (the time from the end of a Failed stop up to the next trial), and *Post-hit processing* (the time from the end of a correct stop or correct go up to the next trial, excepting *Post-error-processing* trials). *Post-hit-processing* and *Post-error-processing*, although not typically used as SST performance metrics, were here considered to assess the neural correlates of predicted learning update processes occurring following the reaction phase, including outcome monitoring and adjustment (Berlin and Lee, 2019, Chevrier and Schachar, 2010). *Baseline/Response Preparation* corresponded to intervals preceding the presentation of the cue. Note that *Incorrect Go* (go trials in which participants pressed the wrong-side button) or *Omission Go* trials were not included in the model because were rare events and not all the participants committed those errors.

For the second-level analysis, we applied a random effects (RFX) analysis at the group level to investigate the task-related brain responses in relation response preparation periods (*Correct Go + Successful Stop + Failed Stop + Inter-trial Interval > Response Preparation*; RFX, *t*(39) = 4.22, *p*-FDR < 0.001) across the whole brain. This whole-brain exploratory analysis aimed to identify regions of significant activity changes related to inhibitory responses during the entire task (localizer approach) and allowed us to identify regions of interest (ROIs) matching those predicted by current models of inhibition and reinforcement learning, namely the basal ganglia and dopaminergic midbrain regions. Supplementary Fig. 1 illustrates the group brain activity map resulting from the contrast between task performance and response preparation periods. Detailed information regarding the identified clusters is provided in Supplementary Table 2.

ROIs for subsequent analyses were defined based on the resulting clusters. Both anatomical and functional criteria, taking the intersection of our RFX significant functional activation map and anatomical boundaries in the dopaminergic midbrain and basal ganglia were used. To distinguish the clusters within the basal ganglia, we applied the MNI-305 Atlas (Evans et al., 1992) from the Neuroimaging & Surgical Technologies (McGill University). Bonferroni correction was set to the minimum necessary threshold (RFX, *t*(39) = 7.21, *P*-Bonferroni = 0.003) to separate our ROIs, namely the caudate and putamen. To identify the midbrain regions, as these are smaller structures and difficult to isolate with fMRI (Chevrier and Schachar, 2010, D’Ardenne et al., 2008, Richter et al., 2020), we used the intersections between our whole-brain maps corrected at a more liberal threshold (RFX, *t*(39) = 4.22, *P-FDR* = 0.001) and two probabilistic atlases of the SN and VTA (Ballard et al., 2011, Murty et al., 2014) from the Adcock Lab (Duke University). We then confirmed the selected clusters by comparing our peak voxel coordinates with previously outlined anatomical landmarks (Ballard et al., 2011, Murty et al., 2014).

*Beta*-values were extracted from the resulting ROIs in the caudate, putamen, VTA, and SN, for three contrasts of interest: successful inhibition (*Successful Stop* > *Correct Go*), failed inhibition (*Failed Stop* > *Correct Go*) (Korucuoglu et al., 2021), and error-processing (*Post-error-processing* > *Post-hit processing*). This analytical strategy is supported by several SST investigations indicating that error-monitoring and post-error behavioural adjustment are encoded during and immediately following inhibition failures (Chevrier & Schachar, 2010).

In the subsequent analysis, we used the Statistical Package for Social Sciences, version 29 (SPSS ®, Chicago, IL, USA) to assess between and within-group differences in our variables as well as their correlational and hierarchical patterns within each group. To analyse between-group differences in task performance, considering the overlap across our variables (*Go RT*, *Post-error RT*, *Omission Go*, *Successful Stopping*, *SSRT*), we used univariate statistics. To test if activation differences between the HC and OCD subgroups in the striatum and midbrain depended on the inhibition phase, we used the Mixed Repeated Measures ANOVA, followed by the *post hoc* independent *t*-tests. We tested one first-order model and two second-order models with ROI (VTA, SN, caudate, and putamen) and condition (successful inhibition, failed inhibition, and error-processing) as within-subjects factors, and group (HC and OCD) as between-subjects factor. Then, we tested correlations between brain activation and a) task performance, in each subgroup, and b) OCD symptoms, in the OCD subgroup. Finally, we conducted a mediation analysis using PROCESS macro (Model 4) for SPSS (Hayes et al., 2020) to test, based on the actor-critic model, the mediation role of the striatum on the relationship between midbrain activation and OCD symptoms. The resulting statistics were FDR-corrected at *P* = 0.05. Because Brain Voyager runs on parametric statistics and most of the variables presented normal distribution (except 2 out of 19 in the OCD group, and 3 out of 16 in the HC group) in the Shapiro-Wilk’s test, we used parametric tests in all the analyses. All the statistical tests were two-tailed.

#### 2.6.2 Functional connectivity

Functional connectivity analysis was conducted using the CONN toolbox (version 22a) in MATLAB 2020b (MathWorks®) (Nieto-Castanon, 2020, Penny et al., 2011, Whitfield-Gabrieli and Nieto-Castanon, 2012), based on the “actor-critic” ROIs defined in our activity analysis, using both anatomical and functional criteria.

The functional and anatomical data underwent standard preprocessing step (Nieto-Castanon, 2020) including realignment with correction of susceptibility distortion interactions, slice timing correction, outlier detection, direct segmentation and MNI-space normalization, and spatial smoothing (4 mm full-width at half-maximum Gaussian kernel). Moreover, functional data were denoised to mitigate potential confounding effects (Nieto-Castanon, 2020). This included regression of signals related to white matter (5 components), CSF (5 components), motion parameters and their first-order derivatives (12 components), ART-based scrubbing in which scans with framewise displacement above 0.9 mm or global BOLD signal changes above 5 s.d. were flagged as potential outliers (153 components in total corresponding to less than 1 % of the analysed data), and accounting for task effects and their first order derivatives (4 components). Finally, linear detrending was also applied, and the subject-specific denoised BOLD signal timeseries were band-pass filtered to eliminate both low-frequency drift effects and high-frequency noise, thus retaining frequencies between 0.008 and 0.09 Hz.

Seed-based connectivity and ROI-to-ROI connectivity matrices were then estimated as the Fisher-transformed bivariate correlation coefficients (Nieto-Castanon and Whitfield-Gabrieli, 2022), defined separately for each pair of ROIs BOLD timeseries or between ROI BOLD timeseries and each voxel BOLD timeseries, respectively. Individual scans were weighted by a boxcar signal characterizing each experimental condition convolved with an SPM canonical hemodynamic response function and rectified. Group-level analyses were performed using a GLM. For each voxel/ROI a separate GLM was estimated, with first-level connectivity measures at this voxel/ROI as dependent variables (one independent sample per subject and one measurement per experimental condition), and groups (OCD and control) as independent variables.

Seed-based connectivity analysis was based on parametric statistics from Gaussian Random Field theory (Worsley et al., 1996) and the results were thresholded using a combination of a cluster-forming *P* < 0.001 voxel-level threshold to initially define clusters of interest from the original statistical parametric maps, and a familywise corrected *P*-FDR < 0.05 cluster-size threshold to select among the resulting clusters, those deemed significant. ROI-to-ROI connectivity analyses were based on functional network connectivity multivariate parametric statistics (Jafri et al., 2008). Results were thresholded using a combination of a familywise corrected *P*-FDR < 0.05 cluster-level threshold to select among all connectivity sets those deemed significant, with a *post-hoc* uncorrected *P* < 0.05 connection-level threshold to help characterize the pattern of individual connections.

## 3 Results

### 3.1 Stop-signal task behavioural performance

OCD participants had slower *Go RT* (HC: 602.95 ± 155.87 *vs.* OCD: 718.47 ± 180.13 ms, *t*(38) = −2.17, *P*-FDR = 0.045), especially on go trials following failed stop trials (*Post-error RT*: HC: 597.2 ± 168.89 *vs.* OCD: 727.7 ± 179.97 ms, *t*(38) = −2.37, *P*-FDR = 0.045). Adherence to the task rules was slightly higher in the HC group as indicated by the rate of *Omission Go* trials (HC: 0.71 ± 1.36 % *vs.* OCD: 2.45 ± 3.33 %, *t*(38) = −2.12, *P*-FDR = 0.045), but was present in both groups as shown by the low number of omissions (< 5 %). In around half of the stop-trials, both HC (51.73 ± 3.05 %) and OCD subjects (55 ± 7.53 %) were able to successfully withhold their response (*Successful Stopping*), while in the other half, they failed to stop, which reflects the effective operation of the staircase procedure. The *SSRT* was within the normal range in the HC group (244.2 ± 36.07 ms) (Williams et al., 1999), while it was increased in the clinical group (272.5 ± 74.89 ms), being consistent with values previously found for adults with OCD (Kang et al., 2013; Menzies et al., 2007). Group comparison revealed neither significant differences in the *SSRT* (*t*(38) = −1.55, *P* = 0.130) nor *Successful Stopping* (*t*(38) = −1.83, *P* = 0.075) scores. This was in part expected considering the experimental homogenization of the SST variables, caused by the adaptive staircase procedure applied during the task implementation. Detailed information regarding the participants’ performance on the SST is presented in Supplementary Table 3.

The present results support the hypothesis that, during inhibition, OCD patients use a distinct learning strategy characterized by slower responses, especially after errors.

### 3.2 Striatum and dopaminergic midbrain recruitment during the SST

**Fig. 2:**
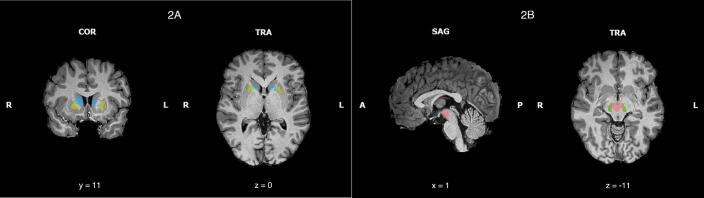
Regions of interest based on the actor-critic model. Regions of interest to investigate inhibitory processes in OCD were defined in the striatum and midbrain using the intersection between our whole-brain activations related to the stop signal task and anatomical boundaries (Araújo et al., 2024, Ballard et al., 2011, Evans et al., 1992, Murty et al., 2014) of the caudate’s head (blue) and dorsal putamen (yellow) (RFX, t(39) = 7.21, P-Bonferroni = 0.003) (2A), ventral tegmental area (pink), substantia nigra (green) (RFX, t(39) = 4.22, P-FDR = 0.001) (2B). Bonferroni correction was set to the minimum threshold that allowed us to isolate these regions of interest from the larger clusters. (For interpretation of the references to colour in this figure legend, the reader is referred to the web version of this article.)

ROI analysis was focused on the basal ganglia and its dopaminergic connections with the midbrain, based on our hypotheses. We first performed whole-brain GLM analysis including the entire sample (HC and OCD) to localize the task-relevant basal ganglia and midbrain regions, which were nevertheless further confirmed with anatomical criteria (for detailed information regarding the strategy to define our relevant ROIs see the Methods section, Supplementary Fig. 1, and Supplementary Table 2). Then, we extracted the *beta*-values from the bilateral caudate’s head, dorsal anterior putamen (Fig. 2A), VTA and SN, the head of the caudate and dorsal anterior putamen (Fig. 2B; Table 1) for our three contrasts of interest: successful inhibition (*Successful Stop > Correct Go*), failed inhibition (*Failed Stop > Correct Go*), and error-processing (*Post-error-processing* > *Post-hit processing*).

**Table 1:** List of striatum and midbrain regions used for region of interest analysis, based on our hypothesis.

Region of interest	Peak x^a^	Peak y^a^	Peak z^a^	Nr of voxels
Caudate’s head bilateral	18	16	0	2280
Dorsal Putamen bilateral	19	15	0	1616
Lateral VTA bilateral	7	−15	−9	1376
Substantia nigra bilateral	10	−22	−10	822

a Coordinates are indicated in MNI space.

### 3.3 Effect of inhibition phase on OCD activation changes in the striatum and midbrain

To test if activation differences between HC and OCD groups in the midbrain and striatum were contingent on each inhibition phase, we used the Mixed Repeated Measures ANOVA. Model 1, with phase (successful inhibition, failed inhibition, and error-processing) and ROI (caudate, putamen, VTA, and SN) as within-subjects factors, and group (HC and OCD) as between-subjects factor, showed a significant interaction of group x phase x ROI (*F*(6,33) = 2.757, *P* = 0.039; Supplementary Table 4.1).

**Fig. 3:**
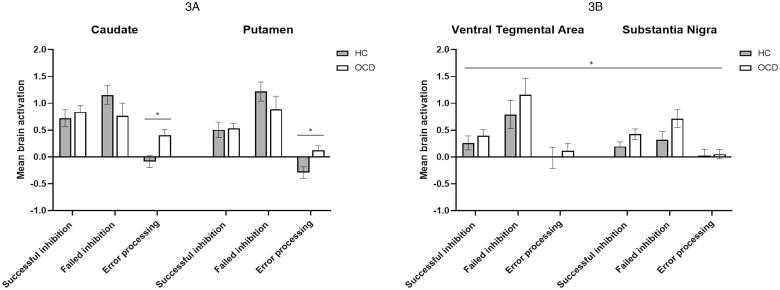
Mean brain activations (beta-values) in the striatum (3A) and midbrain (3B) during inhibition phases. Error bars represent the standard error of the mean. GLM analysis in the striatum revealed a group x phase interaction (*Z*(2,37) = 4.106, *P* = 0.024), which was driven by error-processing-related OCD hyperactivation in the caudate (*t*(38) = −3.201, *P-*FDR = 0.018) and putamen (*t*(38) = −2.928, *P*-FDR = 0.018) (see * symbol). In the midbrain, there was a significant group effect (*Z*(1,38) = 4.376, *P* = 0.043), resulting from the overall contribution of the ventral tegmental area and substantia nigra activations during all task phases (see * symbol). HC: healthy control group; OCD: obsessive–compulsive disorder group.

We then performed two second-order models to further investigate and identify brain activity differences separately in the ROIs corresponding to the “actor” (caudate and putamen; Model 2; Supplementary Tables 4.2 and 4.4, Fig. 3A), and the “critic” (VTA and SN; Model 3; Supplementary Tables 4.3 and 4.5, Fig. 3B), again considering each inhibition phase. Model 2 revealed a significant group x phase interaction (*Z*(2,37) = 4.106, *P* = 0.024), which according to *post-hoc* analysis, was driven by significantly increased error-processing-related caudate (*t*(38) = −3.201, *P*-FDR = 0.018) and putamen (*t*(38) = −2.928, *P*-FDR = 0.018) activation, in the OCD group. Model 3 revealed a significant effect of group alone (*Z*(1,38) = 4.376, *P* = 0.043) and no significant interactions between the group and the other factors, indicating that this difference resulted from an overall effect of both midbrain regions during all task phases being examined.

These results suggest an OCD profile characterized by hyperactivation in the “actor-critic” regions, which was a) contingent to error-processing phases, in the striatum, and b) consistent across task phases, in the midbrain.

### 3.4 Associations between error-processing activity in the striatum and task strategy

To characterize the relationship between the task strategy applied by each group and the underlying neural responses in the “actor” domain (corresponding to the striatum), we performed neurobehavioural correlations with the caudate’s and putamen’s activty variables related to error-processing, because this was the inhibition phase revealing between-group differences. We found correlations between longer response times and error-processing putaminal activity in the HC group (*Go RT*: *r* = 0.511, *P*-FDR = 0.023; *Post-error RT*: *r* = 0.546, *P*-FDR = 0.023), and with error-processing caudate activity in the OCD group (*Go RT*: *r* = 0.523, *P*-FDR = 0.018; *Post-error RT*: *r* = 0.510, *P*-FDR = 0.018) (see Supplementary Table 5).

These results point to a disruption in the relationship between caudate and putaminal activity in determining response inhibition styles of individuals with OCD.

### 3.5 Associations between error-processing activity in the striatum-midbrain and OCD symptoms

The OCD group exhibited a correlation between increasing error-processing activity in the caudate and both decreased global OCD symptoms (*r* = −0.547, *P*-FDR = 0.015) and compulsions (*r* = −0.673, *P*-FDR = 0.004), as measured by the *Y-BOCS-2* (see Supplementary Table 6). Correlations between putaminal error-processing activity and symptoms were all non-significant. To account for the intermediate role of the “actor” in the relationship between the “critic” and action selection, we performed partial correlations between error-processing activity in the midbrain (VTA and SN) and OCD symptoms, controlling for the effect of the caudate. This analysis revealed significant positive correlations between activity in the VTA and both global OCD symptoms (*r* = 0.470, *P*-FDR = 0.049) and obsessions severity (*r* = 0.595, *P*-FDR = 0.018) (see Supplementary Table 6).

### 3.6 Mediation effect of the caudate in the relationship between the ventral tegmental area and OCD symptoms

**Fig. 4:**
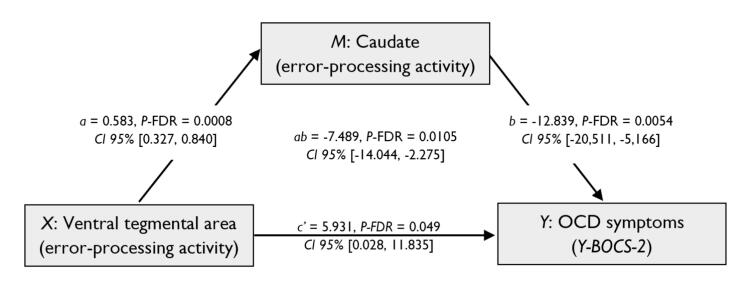
Effect of the relationship between midbrain and striatal error-processing activity on OCD symptom severity. Serial multiple mediation model showing significant direct positive and indirect negative effects of the ventral tegmental area on OCD symptoms severity mediated by the caudate. Numbers represent unstandardized coefficients. Y-BOCS-2: Yale-Brown Obsessive-Compulsive Scale – Second Edition; *X*: predictor variable; *M*: mediator; *Y*: outcome variable; *a*: effect of *X* on *Y*; *b*: effect of M on Y; *ab*: indirect effect of *X* on *Y*; *c’* direct effect of *X* on *Y*; CI: confidence interval.

Based on the actor-critic framework and our previous results, mediational analysis for symptom severity identified two distinct error-related pathways in the VTA leading to direct increases (*Direct effect*: 5.931, *P-*FDR = 0.049, *CI 95 %* [0.028, 11.835]) and caudate-mediated decreases (*Indirect Effect*: −7.489, *P*-FDR = 0.0079, *CI 95 %* [-14.044, −2.275]) in OCD symptoms (Fig. 4). The indirect pathway explained 45.43 % of symptom variance. The total effect was non-significant, which is explained by the opposing direct and indirect pathway effects.

The present model provides evidence for the relevance of two distinct pathways originating in the midbrain leading to increases and decreases in OCD symptoms.

### 3.7 OCD functional connectivity changes in the actor-critic network

To investigate connectivity differences between OCD and HC groups, we considered the “actor-critic” regions (VTA, SN, caudate, and putamen) and analysed functional connectivity within the network nodes (ROI-to-ROI connectivity) as well as between the regions of this network and the whole brain (seed-based connectivity).

**Fig. 5:**
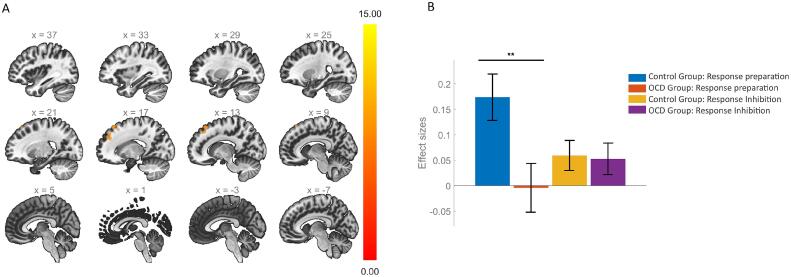
Whole-brain functional connectivity analysis when contrasting the OCD and control group and looking for differences either during response preparation or inhibition and considering the VTA as seed region. Individuals with OCD exhibited reduced connectivity between the VTA and a cluster centered in the prefrontal cortex (coordinates: *x*, *y*, *z* = 13, 40, 42; *F(2,37)* = 10.69, *P*-FDR = 0.0002; A), specifically during response preparation periods (*t*(38) = 4.542, *P* < 0.001; B). The colour bar represents the strength of the *F*-statistic when evaluating how much connectivity differs between OCD and healthy subjects either during response preparation or response inhibition periods. The differences in functional connectivity between the seed region and the rest of the brain are displayed at a voxel-level threshold of *P* = 0.001 and a cluster-size threshold of *P*-FDR = 0.05.

ROI-to-ROI analysis revealed no significant differences between groups within the “actor-critic” network, neither during response preparation nor response inhibition. Regarding seed-based connectivity, OCD participants exhibited reduced connectivity during response preparation periods between the VTA and a cluster centered in the prefrontal cortex (coordinates: *x*, *y*, *z* = 13, 40, 42), encompassing regions in the right frontal pole and right superior frontal gyrus (*F(2,37)* = 10.69, *P*-FDR = 0.0002; Fig. 5).

None of the remaining regions used as seed (caudate, putamen, and SN) showed significant differences between groups in the functional connectivity trajectories for the tested threshold (cluster: *P*-FDR < 0.05; voxel: *P* < 0.001). Nevertheless, an exploratory analysis using more liberal voxel-level thresholds (*P* < 0.02 for SN and *P* < 0.04 for caudate), showed decreased functional connectivity, mostly during response preparation periods, for OCD, when using the SN and the caudate as seed regions (Supplementary Fig. 2). We found decreased connectivity in OCD between the SN and two clusters, one centered in the cingulate cortex (coordinates: *x*, *y*, *z* = −8, 22, 22) involving regions in both the posterior and anterior divisions of the cingulate gyrus, and thalamus (*F(2,37)* = 10.73, *P*-FDR = 0.0002), and other cluster centered on the anterior prefrontal cortex (coordinates: *x*, *y*, *z* = 18, 28, 40) including the bilateral superior frontal gyrus, left frontal pole, left paracingulate gyrus (*F(2,37)* = 11.51, *P*-FDR = 0.0002); and between the caudate and a cluster centered in the cerebellum (coordinates: *x*, *y*, *z* = 2, −52, −40; *F(2,37)* = 20.69, *P*-FDR = 0.000001).

## 4 Discussion

We addressed the question of whether the actor-critic framework and its neural implementation can elucidate the dynamic nature of neurobehavioural inhibitory impairments in adults with OCD. We compared phase- and domain-dependent performance strategies of patients and control subjects, based on an actor-critic explanatory framework. Consistent with our main hypothesis, we identified unique neural patterns in the “actor-critic” network in individuals with OCD, reflecting specific behavioural inhibition strategies and influencing symptom severity. In this context, a major novel finding was the identification of a striking dichotomy in OCD characterized by hypoconnectivity and hyperactivation in the VTA and SN (the “critic”), along with excessive behavioural slowness associated with error-processing-specific hyperactivation in the caudate’s head (the “actor”). We will now discuss our major findings in detail.

Before engaging on the SST, both groups were instructed that optimal performance required a balance between stopping on stop-trials and being fast on others and that there was a trade-off between these two goals (“speed-accuracy trade-off”). Despite that instruction, individuals with OCD were slower due to difficulties in reaction speed modulation for performance optimization (Norman et al., 2019, Thakkar et al., 2008). Moreover, task connectivity between the “actor-critic” nodes and cortical regions was reduced in this clinical group, which suggests an impairment in value-learning and action control (Balleine and O’Doherty, 2010, Haber and Knutson, 2010, Hollerman and Schultz, 1998) underlying flexible strategy adaptation (Everitt and Robbins, 2005, Schönberg et al., 2007). Connectivity within the striatum-midbrain network remained intact, indicating that, similar to other neurodevelopmental disorders (Abbott et al., 2018), OCD predominantly affects long-range connectivity. Specifically, we observed hypoconnectivity, during response preparation phases, between the VTA and right superior frontal gyrus, including dorsolateral areas involved in cognitive control and executive functions (Li et al., 2013). A complementary sensitivity analysis demonstrated additional nodes of reduced connectivity in the “actor-critic” network. Those included connections between the SN and salience processing regions (posterior and anterior divisions of the cingulate gyrus, and thalamus, and anterior prefrontal cortex) previously implicated in instances of increased error-related negativity in OCD, and between the caudate and cerebellum. These findings notably align with recent studies showing frontolimbic and cerebellar hypoconnectivity in individuals with OCD performing the SST (Hampshire et al., 2020, Van Velzen et al., 2015). Further, they suggest that the midbrain influences and/or is influenced by cortical regions in the pathophysiology of OCD, predominantly affecting response preparation phases where proactive control is expected to be engaged. In optogenetically-manipulated mice, Xue et al. (2022) found dopaminergic projections from the SN to the striatum and orbitofrontal cortex involved in OCD-like repetitive behaviours. In humans, it has been suggested, though not yet proven, that dopamine neurons projecting from the midbrain to other brain regions contribute to the neurobiology of OCD (Pagliaccio et al., 2023). Here, we provide evidence for the functional relevance of an OCD “extended circuit” (Menzies et al., 2008) in which midbrain communication with cortical regions during proactive inhibition is reduced. Interestingly, resting-state connectivity between frontoparietal, salience, and default-mode networks also appears to be reduced in OCD (Gürsel et al., 2018), highlighting that hypoconnectivity may occur both in the absence of task demands and during specific cognitive processes.

In terms of task-related neural activity, we observed hyperactivation in the clinical group, resulting from the contribution of both midbrain regions across all inhibition phases, in combination with error-processing-specific striatal hyperactivation. This indicates that enhanced activity in the VTA and SN was a consistent abnormality in OCD during response inhibition, encompassing all task requirements (response or error-processing) and trial outcomes (successful or failed). Over functioning in the midbrain regions, corresponding to the “critic” module, aligns with the prevalent tendency in OCD patients to exhibit persistent hypervigilance towards potentially harmful stimuli (Pitman, 1987, Salkovskis, 1999). Moreover, hyperactive self-monitoring mechanisms can trigger the pervasive sense of incompleteness (“not just right experience”), leading to cognitive rigidity and performance deficits (Norman et al., 2019, Pitman, 1987) mediated in the dorsal striatum (the “actor”), as indicated by our results in this region. Particularly, we found a remarkable dissociation during error-processing with caudate and putaminal deactivation in healthy subjects, and (hyper)activation in individuals with OCD. The corresponding behavioural output in the clinical group was a caudate-correlated deceleration in reaction speed.

Error-processing corresponds to the period when individuals are expected to adjust their strategies in response to task demands and current outcomes (Verbruggen and Logan, 2008). Chevrier and Schachar (2010) proposed that midbrain and striatal deactivation during these phases is critical for flexible and efficient behavioural adjustment. Thus, while striatolimbic deactivation in healthy individuals is thought to reflect an adaptive “reset” of the cognitive strategy (Chevrier, 2010), our results propose that hyperactive caudate-mediated error-signaling in OCD causes overliance on cautious response styles. Thus, although OCD participants in the present study should rationally know that committing errors was inevitable (as indicated by the instructions), their evaluation of these events may have been biased, leading to the adoption of egodystonic “safety-seeking” strategies (Apergis-Schoute et al., 2017).

Overcautious response tendencies have been previously identified in OCD during response inhibition (Norman et al., 2019) and planning (Vaghi et al., 2017b). Clinically, this counterproductive cognitive style often manifests in “obsessive slowness”, a pattern of slow motor performance that interferes with the daily activities of affected individuals (Rachman, 2003). Thus, our findings align with previous reports of excessive slowness in OCD, and add that overfunctioning in the “actor-critic” brain regions interfering with post-error down-regulation processes may underly such “harm-avoidant” profiles. This rationale also provides a potential neurobiological explanation matching findings of ineffective goal-directed behaviour in OCD patients due to excessive state transition uncertainty during decision-making (Fradkin et al., 2020).

The dichotomy between the caudate and putamen in executing goal-directed and habitual actions has been extensively discussed. Our results suggest that failures to deactivate both systems during error-processing are involved in OCD. However, contrarily to healthy subjects, the caudate alone determined the performance output in the clinical group, highlighting that individuals with OCD may disproportionately rely on goal-directed control to modify their behaviour following negative outcomes. This view aligns with recent findings of enhanced goal-directed learning observed in OCD patients in response to loss outcomes (Voon et al., 2015). It is also consistent with the study from Fradkin et al. (2020), who demonstrated that when goal-directed behaviour is driven by uncertainty, it becomes computationally expensive, leading to the emergence of habits as optimal actions. Robbins et al. (2019) proposed that, in some situations, individuals with OCD exhibit overly deliberate or exploratory goal-directed behaviour, especially in the context of an overall narrowing of goals. Similarly, evidence in the present study contradicts the notion of a strict habitual dominance in OCD and supports that imbalances in goal-directed and habitual activities are domain-specific and contextual/value-modulated. We also propose that the midbrain, due to its relationship with functionally reciprocal regions in the ventral and dorsal striatum, is strategically positioned to influence the arbitration between goal-directed and habitual systems.

Further emphasizing that inhibition impairments in OCD are complex and differently affect narrower subdomains, we found that the final inhibitory output, as assessed by the *SSRT* scores, was intact in the clinical group. Together, results from our neurobehavioural analysis suggest that OCD patients employ less efficient “safety-seeking” strategies, driven by error-processing-specific caudate hyperactivation, to achieve the same outcomes as healthy individuals. Therefore, it is comprehensible why research designs that rely on a single index of inhibitory learning and related neural functioning fail to capture the domain-dependency of OCD-related changes, leading to contradictory results (Carlisi et al., 2017, Menzies et al., 2007, Norman et al., 2019). Conversely, the idea of dysfunctional neural pathways dynamically interacting with each other and the environment to determine performance outcomes in OCD crucially reconciles the co-occurrence of distinct neural and behavioural inhibition patterns in OCD.

Correlations with *Y-BOCS-2* symptoms revealed an association between error-processing activity in the striatum and midbrain, and the severity of compulsions and obsessions, respectively. These links remarkably align with clinical characterizations of OCD, and directly implicate the “actor-critic” regions, and corresponding action selection and value-based functions, in its core symptoms. Accordingly, we also identified two opposing error-related pathways originating in the VTA leading to direct increases and caudate-mediated decreases in OCD symptomatology. Thus, hyperactive error-signaling in the midbrain may simultaneously reinforce the repetition of obsessions and compulsions and trigger the striatum to implement down-regulation mechanisms. The present hypothesis is consistent and may explain findings from Pagliaccio et al. (2023) on dopaminergic alterations in children with OCD. These authors observed enhanced neuromelanin-MRI signal (a proxy for dopaminergic function) in the VTA and SN pars compacta, along with an intriguing inverse relationship between this biomarker and lifetime symptoms. Although it operates at a different analytical level, the opponency we found between the VTA and caudate offers an explanatory pathway for Pagliaccio et al. results, in which abnormally enhanced dopaminergic function leads to symptom attenuation. We also provide support for the notion that excessively high dopamine levels lead to detrimental outcomes but may also provide compensatory benefits (Pagliaccio et al., 2023).

Evidence that dopamine dysregulation in OCD may have paradoxical effects first came from clinical settings (Jenike, 2004). While around half of OCD patients do not respond to first-line treatments and require augmentation with dopamine receptor antagonists, some of these medications can induce obsessional effects (Maina et al., 2008, McDougle et al., 2000). By blocking compulsive symptoms mediated by the dorsal striatum, antipsychotic agents may uncover obsessional symptoms linked to the ventral striatum. In the same vein, we propose that antagonistic relationships between the VTA and caudate may influence symptom severity in OCD and complicate treatment due to dynamic adaptation and feedback loop mechanisms. This idea aligns with the observation that OCD patients typically respond to low doses of atypical antipsychotics, whereas higher doses often cause side effects without additional therapeutic benefits. Conversely, some OCD subgroups respond to psychostimulant (pro-dopaminergic) therapy. Thus, by providing a comprehensive framework, our results underscore that the key to antipsychotic potentiation for OCD lies in achieving an optimal balance of dopaminergic activity.

Future studies using direct measures of dopaminergic function, as conducted by Pagliaccio et al. (2023) and Denys et al. (2004), will be essential to elucidate the exact nature of the alterations in neurotransmitter systems involved in OCD. Another limitation of our study was the inclusion of an exclusively male sample, entailing that the present findings only apply to this subgroup. Given the heterogeneity of OCD, particularly sex-dependent differences in clinical characteristics and comorbidities (Taylor, 2011), generalization to broader patient populations requires further testing, preferably in larger samples. Finally, the utility of the actor-critic model in elucidating the neurobiological underpinnings of OCD and other impulsive/compulsive disorders requires validation with computational modeling techniques.

### 4.1 Conclusion

In conclusion, we identified, in OCD patients, a dichotomous neural pattern of decreased connectivity and increased activity in the “actor-critic” regions associated with symptom severity and alterations in response inhibition. Our novel approach integrates previous explanatory models of OCD, such as impaired response inhibition, dysfunctional learning, and aberrant error monitoring, incorporating as well cognitive, metacognitive, and symptom constructs. While the actor-critic framework has previously shown its value in investigating the learning mechanisms of animals and healthy humans (Araújo et al., 2024, Jin and Costa, 2015, O’Doherty et al., 2004, Solié et al., 2022), the profile we identified here in individuals with OCD was not described before. We demonstrated that dysfunctional inhibition, driven specifically by over functional error-processing mechanisms in the midbrain and striatum and affecting proactive inhibition phases, is crucial in OCD and its core symptoms. By broadening the regions implicated in the “OCD circuit” to include the dopaminergic midbrain and its connections, our findings may inform the development of neurobiologically-based neuromodulation therapies and validate the current ones. Whether hyperactive error-processing during inhibition is specific to OCD or represents a transdiagnostic impairment affecting other psychiatric conditions could be assessed if more researchers incorporate such learning theory principles in the experimental testing of functional impairments. We, thus, advocate for the scientific utility of the actor-critic model in investigating the neural underpinnings of neuropsychiatric disorders defined by impulsivity and compulsivity.

## Author contributions

A.A., I.C.D. and M.C.B. designed the experiment and set the analysis plan. A.A., T.S. and I.C.D. analysed the data. All authors contributed to interpretation of the data. A.A. wrote the initial draft of the manuscript. All authors reviewed the manuscript.

## CRediT authorship contribution statement

**Ana Araújo:** Writing – review & editing, Writing – original draft, Visualization, Formal analysis, Conceptualization. **Isabel C. Duarte:** Visualization, Validation, Methodology. **Teresa Sousa:** Writing – review & editing, Visualization, Validation, Methodology, Formal analysis. **Sofia Meneses:** Writing – review & editing, Validation, Investigation. **Ana T. Pereira:** Writing – review & editing, Supervision, Investigation. **Trevor Robbins:** Writing – review & editing, Validation, Supervision, Investigation. **António Macedo:** Writing – review & editing, Validation, Supervision, Investigation. **Miguel Castelo-Branco:** Writing – review & editing, Visualization, Supervision, Project administration, Investigation, Funding acquisition, Data curation, Conceptualization.

## Funding

This work was supported by the Portuguese Foundation for Science and Technology (FCT) (grants: UID/04950B/2020, UID/04950P/2020, UID/4950/Base&Prog/2025-2029, DSAIPA/DS/0041/2020, PTDC/PSI-GER/1326/2020, 2022.04701.PTDC). FCT also funded a Ph.D. scholarship to AA (2020.08114.BD) and an individual contract to TS (2021.01469.CEECIND).

## 2.7 Data availability

The datasets generated and/or analysed during the current study are not publicly available due to ethical restrictions but are available from the corresponding author upon reasonable request.

## Declaration of competing interest

The authors declare the following financial interests/personal relationships which may be considered as potential competing interests: TR is a consultant for Cambridge Cognition and Supernus and editorial honoraria from Springer-Nature and Elsevier and he has a research grant from Shionogi Co.

## Data Availability

Data will be made available on request.
