# Supplementary material for: “Actor-critic” dichotomous hyperactivation and hypoconnectivity in obsessive–compulsive disorder
Source: Neuroimage Clin. 2024 Dec 31;45:103729. doi: 10.1016/j.nicl.2024.103729 (PMC11762915; doi:10.1016/j.nicl.2024.103729)
Supplement: Supplementary Data 1 [file mmc1.docx]

**Supplementary material**

**Supplementary Table 1 Participants’ demographic and clinical characteristics.**

|  | HC  (*n* = 21)  *Mean* (*SD*) | OCD  (*n* = 19)  *Mean* (*SD*) |
| --- | --- | --- |
| Gender, n (male:female) | 100:0 | 100:0 |
| Age (years) | 28.67 (9.03) | 31.68 (9.35) |
| Education (years) | 15.33 (2.35) | 13.16 (3.01) |
| IQ-Full Scale | 125.7 (11.49) | 108.44 (14.23) |
| IQ-Verbal | 126.65 (11.45) | 112.38 (14.1) |
| IQ-Performance | 118.8 (10.9) | 100.38 (15.45) |
| *Clinical Measures* | | |
| MINI* | 0 | 12 |
| Depression |  | 4 |
| Panic disorder |  | 3 |
| Agoraphobia |  | 1 |
| Social phobia |  | 3 |
| Generalized anxiety disorder |  | 1 |
| DSM-5^a^ |  | 7 |
| Tic disorder (related to OCD) |  | 3 |
| Specific Phobia |  | 2 |
| Trichotillomania (hair-pulling disorder) |  | 1 |
| Excoriation (skin-picking) disorder |  | 1 |
| *Medication Status* | 0 | 18 |
| Antidepressants |  | 18 |
| Antipsychotics |  | 5 |
| Lamotrigine |  | 1 |
| Memantine |  | 1 |
| Y-BOCS-II total |  | 25.26 (5.84) |
| Y-BOCS-II obsessions |  | 12.84 (2.75) |
| Y-BOCS-II compulsions |  | 12.42 (3.66) |
| Y-BOCS-II Insight |  | 2.05 (0.71) |

HC: healthy control group; OCD: obsessive-compulsive disorder group; *SD*: standard deviation; IQ: intelligence quotient; MINI: Mini-International Neuropsychiatric Interview; Y-BOCS-2, Yale-Brown Obsessive Compulsive Scale – Second Edition. ^a^Refers to past comorbidity. Some of the participants were experiencing symptoms related to the reported diagnostic entities at the time of the study, but these were subclinical and/or secondary obsessive-compulsive disorder symptoms.

**Supplementary Figure 1 Whole-brain activations related to the stop-signal task.**


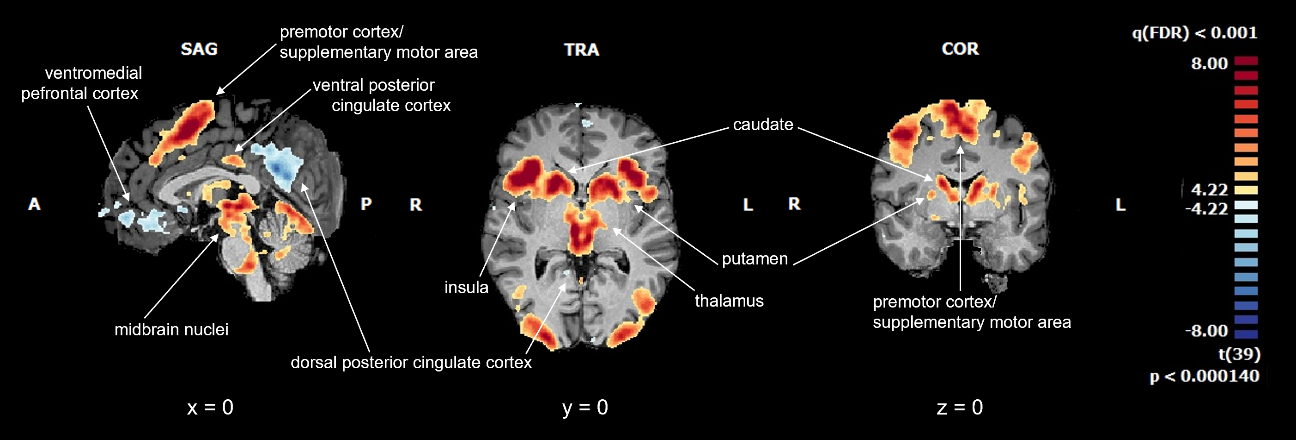


The presented maps were extracted from the contrast between the task and baseline/response preparation (*Correct Go + Successful Stop + Failed Stop + Inter-trial Interval* > *Baseline*/*Response Preparation*, RFX, *t*(39) = 4.22, *P*-FDR < 0.001), including all the participants. Regions in yellow-orange tones and regions in blue tones represent respectively positive and negative changes in brain activity during task performance in relation to response preparation. The presented brain map shows activity changes related to the task performance in regions implicated in action planning and inhibition (ventromedial prefrontal cortex, premotor cortex, premotor and supplementary motor areas, striatum, and thalamus), reward-learning (midbrain nuclei, namely a large cluster including the VTA and SN), and salience (insula). There is also deactivation of the default mode network regions (ventromedial prefrontal cortex and posterior cingulate cortex), which is consistent with the attention shift to external cues imposed by the task.

**Supplementary Table 2 List of significant clusters resulting from our whole-brain analysis**

|  | MNI coordinates (peak) | | |  |  |  |
| --- | --- | --- | --- | --- | --- | --- |
| Region | ***x*** | ***y*** | ***z*** | **Number of voxels** | ***t*** | ***P*** |
| Supramarginal gyrus R | 59 | -44 | 31 | 17824 | 8.244141 | < 0.000001 |
| Anterior PFC/ broca opercularis R/premotor/ SMA/ insula/ supramarginal gyrus L/ basal ganglia/ midbrain | -32 | 22 | 6 | 154801 | 11.403395 | < 0.000001 |
| Angular gyrus R | 51 | -72 | 28 | 2361 | -6.422369 | < 0.000001 |
| Fusiform gyrus R/ secondary visual cortex R/ cerebellum | 41 | -57 | -32 | 41791 | 9.750028 | < 0.000001 |
| Primary sensory cortex R | 41 | -18 | 17 | 417 | -5.787915 | 0.000001 |
| Cerebellum R | 40 | -77 | -40 | 1031 | -5.981069 | 0.000001 |
| Sensory associative cortex R | 24 | -40 | 66 | 1294 | -6.534869 | < 0.000001 |
| Posterior cingulate cortex | -6 | -55 | 7 | 15306 | -9.998752 | < 0.000001 |
| Visual associative cortex R | 7 | -89 | 40 | 2800 | -6.444586 | < 0.000001 |
| Ventral posterior cingulate cortex | 8 | -31 | 28 | 2307 | 8.014505 | < 0.000001 |
| Anteromedial PFC | -8 | 31 | -13 | 5872 | -6.062046 | < 0.000001 |
| Secondary visual cortex L | -10 | -102 | 18 | 1991 | -6.150981 | < 0.000001 |
| Frontal eye fields L | -22 | 32 | 47 | 2367 | -5.994769 | 0.000001 |
| Visual associative cortex L/ fusiform gyrus L/ cerebellum | -32 | -56 | -33 | 25125 | 10.378153 | < 0.000001 |
| Hippocampus L | -18 | -11 | -22 | 584 | -5.473027 | 0.000003 |
| IFC/pars orbitalis L | -32 | 38 | -14 | 2318 | -7.222793 | < 0.000001 |
| Angular gyrus L posterior | -44 | -78 | 28 | 8575 | -9.269484 | < 0.000001 |
| Anterior PFC L | -39 | 42 | 33 | 2778 | 7.602777 | < 0.000001 |
| Angular gyrus L anterior | -57 | -44 | 32 | 1188 | 7.464706 | < 0.000001 |
| Fusiform gyrus L | -59 | -46 | -6 | 884 | -6.062812 | < 0.000001 |
| Medial temporal gyrus L | -63 | -11 | -20 | 777 | -5.396110 | 0.000004 |

Listed cluster resulted from our whole-brain RFX analysis (RFX, *t*(39) = 4.22, *P*-FDR < 0.001; cluster threshold = 50 voxels) when contrasting the task with baseline/response preparation (*Correct Go* + *Successful Stop* + *Failed Stop* *+ Inter-trial Interval* > *Baseline*/*Response Preparation*). R: right; L: left; PFC: prefrontal cortex; SMA: supplementary motor area; IFC: Inferior Frontal gyrus.

**Supplementary Table 3 Performance on the stop-signal task and between-group comparison**

|  | HC  (*n* = 21)  *Mean* (*SD*) | OCD  (*n* = 19)  *Mean* (*SD*) | *Statistics* | *df* | *P* |
| --- | --- | --- | --- | --- | --- |
| Omission Go | 0.71 (1.36) % | 2.45 (3.33) % | *t* = -2.12 | 38 | **0.045**^a^ |
| Go RT | 602.95 (155.87) ms | 718.47 (180.13) ms | *t* = -2.17 | 38 | **0.045**^a^ |
| Post-error RT | 597.2 (168.89) ms | 727.7 (179.97) ms | *t* = -2.37 | 38 | **0.045**^a^ |
| SSRT | 244.2 (36.07) ms | 272.5 (74.89) ms | *t* = -1.55 | 38 | 0.130 |
| Successful Stopping | 51.73 (3.05) % | 55 (7.53) % | *t* = -1.83 | 38 | 0.075 |

Independent samples *t*-tests revealed a univariate effect of the group on the percentage of Omission Go trials, Go RT, and Post-error RT, and (^a^FDR-corrected at *P* = 0.05). Considering the artificial homogenization of Successful Stopping and SSRT scores, caused by the adaptive staircase procedure applied during the task implementation, these variables were not included for FDR-correction. HC: healthy control group; OCD: obsessive-compulsive disorder group; *SD*: standard deviation; *df*: degrees of freedom; Go RT: mean reaction time on go trials; Post-error RT: mean reaction time on trials after failed stop; SSRT: stop signal reaction time.

**Supplementary Table 4.1 Effect of inhibition phase on OCD activation changes in the striatum and midbrain (model 1)**

|  | *df* | *Error df* | *Z* | *P* |
| --- | --- | --- | --- | --- |
| Group | 1 | 38 | 2.157 | 0.150^a^ |
| Group x phase | 2 | 37 | 0.628 | 0.505^a^ |
| Group x ROI | 3 | 36 | 0.869 | 0.433^a^ |
| Group x phase x ROI | 6 | 33 | 2.757 | **0.039**^a^ |

Three-factor repeated measures ANOVA revealed that the effect of group (healthy control [n = 21] *vs.* obsessive-compulsive disorder [n = 19]) depended on the effects of ROI (ventral tegmental area, substantia nigra, caudate and putamen) and phase (successful inhibition, failed inhibition, and error-processing). ^a^Huynh-Feldt corrected.

**Supplementary Table 4.2 Effect of inhibition phase on OCD activation changes in the striatum (model 2)**

|  | *df* | *Error df* | *Z* | *P* |
| --- | --- | --- | --- | --- |
| Group | 1 | 38 | 0.212 | 0.648^a^ |
| Group x phase | 2 | 37 | 4.106 | **0.024**^a^ |
| Group x ROI | 1 | 38 | 0.129 | 0.722^a^ |
| Group x phase x ROI | 2 | 37 | 0.279 | 0.710^a^ |

Three-factor repeated measures ANOVA revealed an effect of group (healthy control [n = 21] *vs.* obsessive-compulsive disorder [n = 19]) x phase (successful inhibition, failed inhibition, and error-processing) in the striatum (caudate and putamen). ^a^Huynh-Feldt corrected.

**Supplementary Table 4.3 Effect of inhibition phase on OCD activation changes in the midbrain (model 3)**

|  | *df* | *Error df* | *Z* | *P* |
| --- | --- | --- | --- | --- |
| Group | 1 | 38 | 4.376 | **0.043**^a^ |
| Group x phase | 2 | 37 | 0.560 | 0.528^a^ |
| Group x ROI | 1 | 38 | 0.004 | 0.947^a^ |
| Group x phase x ROI | 2 | 37 | 0.126 | 0.814^a^ |

Three-factor repeated measures ANOVA revealed a group effect (healthy control [n = 21] *vs.* obsessive-compulsive disorder [n = 19]) for midbrain (ventral tegmental area and substantia nigra) activation during all task phases (successful inhibition, failed inhibition, and error-processing).  ^a^Huynh-Feldt corrected.

**Supplementary Table 4.4 Model 2 *post hoc* comparisons**

| Brain activation  (*beta*-values) | HC  (*n* = 21) | OCD  (*n* = 19) | *t*-test | *Cohen’s d* | 95% CI | |
| --- | --- | --- | --- | --- | --- | --- |
|  |  |  |  |  | **Lower** | **Upper** |
| Caudate  (successful inhibition) | *M* = 0.723  *SD* = 0.729 | *M* = 0.837  *SD* = 0.5 | *t* (38) = -0.574  *P-*FDR = 0.683 | -0.182 | -0.802 | 0.441 |
| Putamen  (successful inhibition) | *M* = 0.505  *SD* = 0.653 | *M* = 0.535  *SD* = 0.391 | *t* (38) = -0.176  *P-*FDR = 0.861 | -0.054 | -0.675 | 0.567 |
| Caudate  (failed inhibition) | *M* = 1.154  *SD* = 0.801 | *M* = 0.767  *SD* = 1.029 | *t* (38) = 1.336  *P-*FDR = 0.378 | 0.423 | -0.207 | 1.048 |
| Putamen  (failed inhibition) | *M* = 1.217  *SD* = 0.819 | *M* = 0.89  *SD* = 1.04 | *t* (38) = 1.107  *P-*FDR = 0.413 | 0.350 | -0.277 | 0.974 |
| Caudate  (error-processing) | *M* = -0.086  *SD* = 0.506 | *M* = 0.404  *SD* = 0.457 | ***t* (38) = -3.201**  ***P-*FDR = 0.018** | -1.014 | -1.669 | -0.347 |
| Putamen  (error-processing) | *M* = -0.291  *SD* = 0.493 | *M* = 0.120  *SD* = 0.384 | ***t* (38) = -2.928**  ***P-*FDR = 0.018** | -0.927 | -1.576 | -0.267 |

*Post hoc* *t*-test comparison of brain activation (beta-values) between groups in each ROIs (caudate and putamen) and task phase (successful inhibition, failed inhibition, and error-processing). *M*: mean; *SD*: standard deviation.

**Supplementary Table 4.5 Model 3 *post hoc* comparisons**

| Brain activation  (*beta*-values) | HC  (*n* = 21) | OCD  (*n* = 19) | *t*-test | *Cohen’s d* | 95% CI | |
| --- | --- | --- | --- | --- | --- | --- |
|  |  |  |  |  | **Lower** | **Upper** |
| Ventral tegmental area  (successful inhibition) | *M* = 0.262  *SD* = 0.596 | *M* = 0.392  *SD* = 0.513 | *t* (38) = -0.738  *P-*FDR = 0.698 | -0.234 | -0.855 | 0.391 |
| Substantia nigra  (successful inhibition) | *M* = 0.194  *SD* = 0.4 | *M* = 0.424  *SD* = 0.437 | *t* (38) = -1.733  *P-*FDR = 0.273 | -0.549 | -1.178 | 0.087 |
| Ventral tegmental area  (failed inhibition) | *M* = 0.7910  *SD* = 1.198 | *M* = 1.158  *SD* = 1.318 | *t* (38) = 0.922  *P-*FDR = 0.698 | -0.292 | -0.914 | 0.334 |
| Substantia nigra  (failed inhibition) | *M* = 0.321  *SD* = 0.71 | *M* = 0.717  *SD* = 0.719 | *t* (38) = -1.751  *P-*FDR = 0.273 | -0.554 | -1.184 | 0.082 |
| Ventral tegmental area (error-processing) | *M* = -0.0148  *SD* = 0.898 | *M* = 0.115  *SD* = 0.594 | *t* (38) = -0.535  *P-*FDR = 0.715 | -0.169 | -0.790 | 0.453 |
| Substantia nigra  (error-processing) | *M* = 0.0225  *SD* = 0.564 | *M* = 0.051  *SD* = 0.369 | *t* (38) = -0.187  *P-*FDR = 0.861 | -0.059 | -0.679 | 0.562 |

*Post hoc* *t*-test comparison of brain activation (beta-values) between groups in each ROI (VTA and SN) and task phase (successful inhibition, failed inhibition, and error-processing). HC: healthy control group; OCD: obsessive-compulsive disorder group; *M*: mean; *SD*: standard deviation.

**Supplementary Table 5 Correlations between brain activity and stop-signal task performance**

| Brain activation  (*beta*-values) | Go RT | | Post-error RT | |
| --- | --- | --- | --- | --- |
|  | **HC**  (*n* = 21) | **OCD**  (*n* = 19) | **HC**  (*n* = 21) | **OCD**  (*n* = 19) |
| Caudate  (error-processing) | *r* = 0.291  *P-*FDR = 0.371 | ***r* = 0.523**  ***P-*FDR = 0.023** | *r* = 0.206  *P-*FDR = 0.371 | ***r* = 0.510**  ***P-*FDR = 0.023** |
| Putamen  (error-processing) | ***r* = 0.511**  ***P-*FDR = 0.018** | *r* = 0.394  *P-*FDR = 0.104 | ***r* = 0.546**  ***P-*FDR = 0.018** | *r* = 0.385  *P-*FDR = 0.104 |

Two-tailed Pearson’s correlations between task performance and brain activity (beta-values). FDR correction was applied within each ROI and considering the two partially overlapping performance parameters included in the analysis. HC: healthy control group; OCD: obsessive-compulsive disorder group. Go RT: mean reaction time on go trials; Post-error RT: mean reaction time on trials after failed stop.

**Supplementary Table 6 Correlations between brain activity and symptoms in the OCD group (*n* = 19)**

| Brain activation  (*beta*-values) | Y-BOCS-2  total score | Y-BOCS-2 obsessions | Y-BOCS-2 compulsions |
| --- | --- | --- | --- |
| Caudate  (error-processing)^a^ | ***r* = -0.547**  ***P* = 0.015** | *r* = -0.267  *P*-FDR = 0.270 | ***r* = -0.673**  ***P*-FDR = 0.004** |
| Putamen  (error-processing)^a^ | *r* = -0.413  *P* = 0.079 | *r* = -0.364  *P*-FDR = 0.126 | *r* = -0.385  *P*-FDR = 0.126 |
| Substantia Nigra  (error-processing)^b^ | *r* = 0.057  *P* = 0.823 | *r* = 0.123  *P*-FDR = 0.943 | *r* = -0.018  *P*-FDR = 0.943 |
| Ventral tegmental area  (error-processing)^b^ | ***r* = 0.470**  ***P* = 0.049** | ***r* = 0.595**  ***P*-FDR = 0.018** | *r* = 0.266  *P*-FDR = 0.286 |

FDR correction was applied within each ROI and considering the partially overlapping effects of the Y-BOCS-2 factors (obsessions and compulsions). **^a^**Two-tailed bivariate Pearson’s correlation. **^b^**Two-tailed partial correlation, controlling for error-processing in the caudate activity. Y-BOCS-2: Yale-Brown Obsessive Compulsive Scale Second Edition.

**
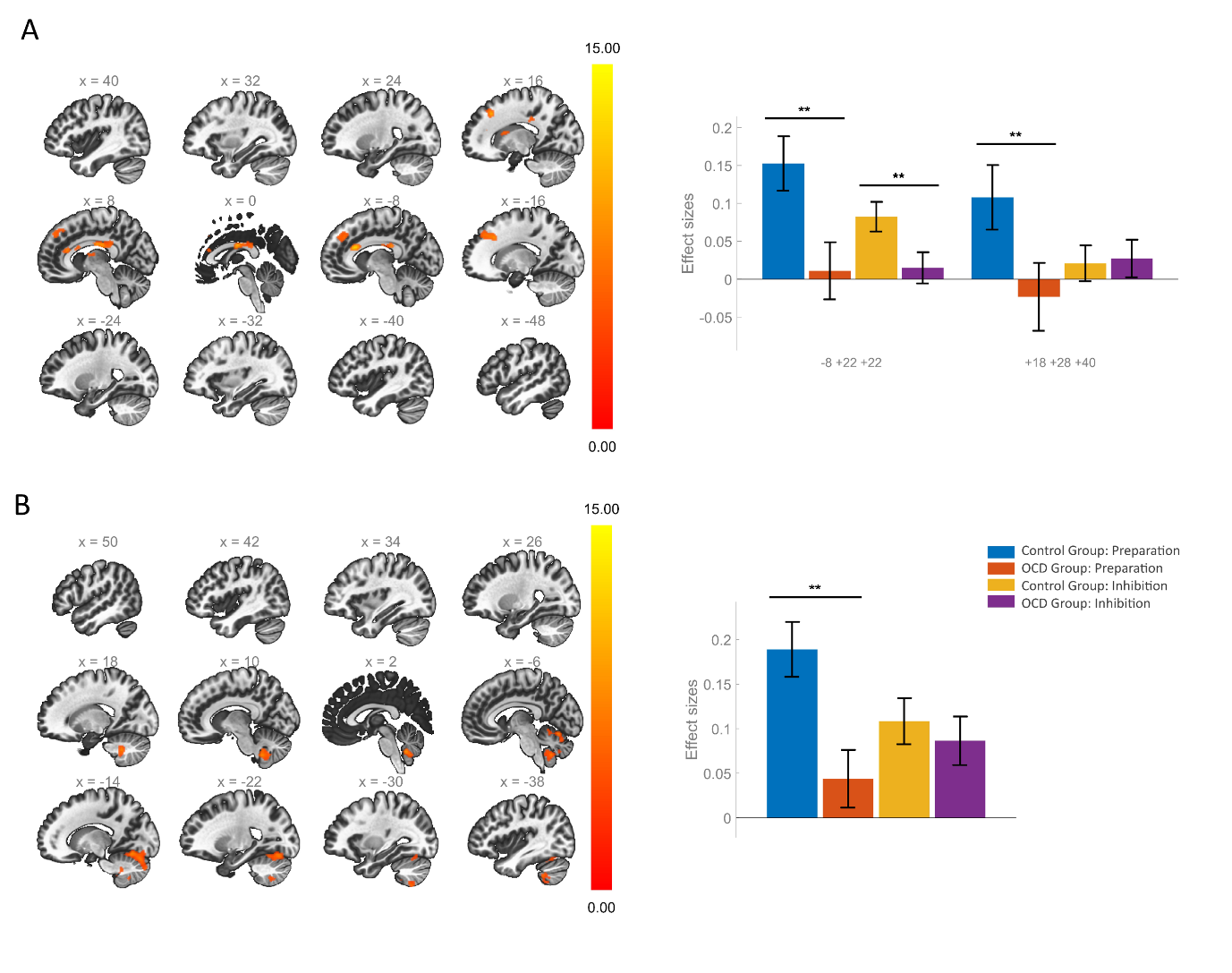
** **Supplementary Figure 2 Whole-brain functional connectivity analysis when looking for potential differences between groups, either during response preparation or inhibition, and considering as seed regions the SN (A) and caudate (B).**

Individuals with OCD exhibited reduced connectivity between the SN (**2A**) and two clusters, one centered in the cingulate cortex (coordinates: x, y, z = -8, 22, 22; *(F*(2,37) = 10.73*, P-*FDR = 0.0002*)*), and other centered on the anterior prefrontal cortex (coordinates: x, y, z = 18, 28, 40; *F*(2,37) = 11.51, *P*-FDR = 0.0002*)*); and between the caudate (**2B**) and a cluster centered in the cerebellum (coordinates: x, y, z = 2, -52, -40; *F*(2,37) = 20.69, *P*-FDR = 0.000001). The colour bar represents the strength of *F*-statistics when evaluating how much connectivity differs between OCD and healthy subjects either during response preparation or response inhibition periods. The differences in functional connectivity between each seed region and the rest of the brain are displayed at a cluster-size threshold of *P*-FDR = 0.05 and a voxel-level threshold of *P* = 0.02 (A) and *P* = 0.04 (B). Note that these results are part of an exploratory analysis using more liberal thresholds than the recommended.
